# Biogenic Synthesis of MnO_2_ Nanoparticles With Leaf Extract of *Viola betonicifolia* for Enhanced Antioxidant, Antimicrobial, Cytotoxic, and Biocompatible Applications

**DOI:** 10.3389/fmicb.2021.761084

**Published:** 2021-11-01

**Authors:** Haibin Lu, Xueyang Zhang, Shakeel Ahmad Khan, Wenqiang Li, Lei Wan

**Affiliations:** ^1^Shunde Hospital, Southern Medical University (The First People’s Hospital of Shunde), Foshan, China; ^2^Stomatological Hospital, Southern Medical University, Guangzhou, China; ^3^Center of Super-Diamond and Advanced Films (COSDAF), Department of Chemistry, City University of Hong Kong, Kowloon, Hong Kong; ^4^Engineering Technology Research Centre for Sports Assistive Devices of Guangdong, Guangzhou Sport University, Guangzhou, China

**Keywords:** MnO_2_ NPs, antimicrobial, biofilm inhibition, antioxidant, cytotoxic

## Abstract

In this study, we propose to synthesize NPs using plant extract containing active biomedical components, with the goal of obtaining NPs that inherit the biomedical activities of the plant. Herein, we report the synthesis of manganese dioxide nanoparticles (VBLE-MnO_2_ NPs) using the leaves extract of *Viola betonicifolia*, in which the biological active plant’s secondary metabolites function as both reducing and capping agents. The synthesized NPs were successfully characterized with different spectroscopic techniques. The antibacterial, antifungal, and biofilm inhibition properties of the synthesized VBLE-MnO_2_ NPs were further explored against a variety of bacteria (Gram-positive and Gram-negative) and mycological species. Additionally, their antioxidant ability against linoleic acid peroxidation inhibition, cytobiocompatibility with hMSC cells, and cytotoxicity against MCF-7 cells were investigated compared to leaves extract and chemically synthesized manganese dioxide NPs (CH-MnO_2_ NPs). The results were demonstrated that the synthesized VBLE-MnO_2_ NPs presented excellent antibacterial, antifungal, and biofilm inhibition performance against all the tested microbial species compared to plant leaves extract and CH-MnO_2_ NPs. Moreover, they also exhibited significant antioxidant potential, which was comparable to the external standard (ascorbic acid); however, it was higher than plant leaves extract and CH-MnO_2_ NPs. Furthermore, the synthesized CH-MnO_2_ NPs displayed good cytobiocompatibility with hMSC cells compared to CH-MnO_2_ NPs. The enhanced antioxidant, antibacterial, antifungal, and biofilm inhibition efficacy as compared to CH-MnO_2_ NPs might be attributed to the synergistic effect of the VBLE-MnO_2_ NPs’ physical properties and the adsorbed biologically active phytomolecules from the leaves extract of *V. betonicifolia* on their surface. Thus, our study establishes a novel ecologically acceptable route for nanomaterials’ fabrication with increased and/or extra medicinal functions derived from their herbal origins.

## Introduction

Antibiotic and antifungal drug resistance in pathogenic bacterial and fungal species have emerged as an alarming threat globally (Khan et al., 2020a). A significant reason behind the antimicrobial drugs not working is the formation of biofilms by these microbials (Khan et al., 2021c). These pathogenic microbials in biofilms form can withstand a thousand dosages of conventional antimicrobial medicines (Khan and Lee, 2020b). Moreover, these pathogenic microbials have also evolved resistance to antimicrobial drugs by developing efflux mechanisms, decreasing the cell wall permeation, modifying the drug targeted sites, etc. (Li and Nikaido, 2009; Khan et al., 2020b). Therefore, these antimicrobial drugs are failing to treat different infectious diseases caused by these pathogenic microbial species. The current death burden due to infectious disease caused by pathogenic microbials is around 0.7 million deaths annually, as per World Health Organization. This can be risen to approximately 10 million annually by 2050 if effective and novel antimicrobial agents are not developed (Khan et al., 2020a; New report calls for urgent action to avert antimicrobial resistance crisis, 2021). In this instance, nanotechnology has emerged and come to the forefront to confront antimicrobial resistance issues by developing nanosized materials.

NPs, among other nanomaterials, are attracting so much attention worldwide because of their unique physical characteristics. Moreover, NPs present excellent biological, electrical, sensing, and optoelectronics applications due to their physical characteristics. National Nanotechnology Initiative has spent more than 27 billion dollars in the United States during the last 10years. Moreover, the European Commission has initiated HORIZON 2020 projects in the nanotechnology sector with around €1.1 billion. In Asia, China and Japan are investing vast amounts of resources and funds for nanoscience and nanotechnology, which resulted in prices rising by 20 percent nearly every year since 2003 (Ciorîță et al., 2020; Home National Nanotechnology Initiative 2021). Metals (Au, Ag, Pt, Pd, Mn, Zn, Cu, etc.) and metal oxides (CuO, MnO, ZnO, NiO, MgO, FeO, Fe_2_O_3_, Cr_2_O_3_, etc.) NPs are widely exploited and investigated for different biological application, such as antibacterial, antimycotic, antibiofilm, antioxidant, and anticancer (Abbasi et al., 2019a,b; Al-Radadi, 2019; Haq et al., 2020; Khan et al., 2020a,b, 2021b; Khan and Lee, 2020a). In contrast to traditional antibacterial and antifungal medicines, these NPs readily penetrate pathogenic microorganisms’ cell walls and membranes due to their nanoscale size. This is a key element in the antimicrobial activities of these NPs (Khan et al., 2020a). Furthermore, MnO_2_ NPs have been much attracted due to their low potential cytotoxicity compared to others.

Generally, NPs are produced through chemical or physical methods. However, both approaches need significant amounts of energy and hazardous chemicals for reduction and capping, and they are not readily scalable (Ciorîță et al., 2020; Khan et al., 2020a,b). A critical issue is that these methods jeopardize the biocompatibility of NPs owing to using dangerous chemicals in the manufacturing process, which remain on the NPs’ interface also after repeated washing. As a result, their biological applicability is jeopardized (Negahdary et al., 2015; Ogunyemi et al., 2019; Ciorîță et al., 2020). Thus, the synthesis of NPs utilizing biological approaches, particularly those based on plants, offers a solution to these established methodologies (Ciorîță et al., 2020; Khan et al., 2020a,b). It has been shown that the phytochemical constituents present in plants, including, alkaloids, polyphenols, flavonoids, and terpenoids, induce the reduction of metal ions and eventual formation of metal NPs (Elbagory et al., 2019; Gharehyakheh et al., 2020). Moreover, it is considered that biogenic plant phytomolecules may enhance their intrinsic properties, such as antioxidant, antibacterial, and anticancer compared to extracts (Chandran et al., 2006; Kumar and Yadav, 2009; Muthukumar et al., 2016). Thus, biogenic synthesis using leaves extract of plants enhances the nanoparticle biocompatibility and is responsible for the synergetic effect (Khan and Lee, 2020a).

In this regard, leaves extract of *Viola betonicifolia* (L.), a species of family *Violaceace*, was used for bioconversion of manganese ions to NPs. *V. betonicifolia* (L.) is naturally available in several countries over the globe, including Pakistan, India, Nepal, Sri Lanka, China, Malaysia, Burma, and Australia (Flora of Pakistan; Rizwan et al., 2019). This plant has been widely exploited as purgative, astringent, diaphoretic, anticancer, antipyretic, and employed for treating various diseases, such as nervous disorders, epilepsy, cough, skin disorders, blood disorders, sinusitis, pharyngitis kidney diseases, bronchitis, and pneumonia (Iqbal and Hamayun, 2005; Rizwan et al., 2019). Various reports demonstrated that the whole leaves extract of *V. betonicifolia* is a rich source of several biogenic phytomolecules, including alkaloids, flavonoids, tannins, phenolic compounds, saponins, and triterpenoids with various biological applications (Muhammad et al., 2012, 2013a). Almost 200 natural active compounds have been identified and isolated from various Viola species (Zhu et al., 2015). Up to now, many plants have been utilized for the biogenic synthesis of MnO_2_ NPs; however, there is no report for the utilization of *V. betonicifolia* (L.). Therefore, here for the first time, we report the biogenic synthesis of MnO_2_ NPs using *V. betonicifolia* (L.) leaves extract. Further considering the leaf leaves extract of *V. betonicifolia* health benefits in the biomedical field, herein, biologically synthesized MnO_2_ NPs were evaluated for antimicrobial, anticancer, and antioxidant activities.

## Materials and Methods

All the chemicals used in this work were purchased from Sigma Chemicals Co (St, Louis, MS, United States) and Merck (Darmstadt Germany) and were analytical grade. The commercially available MnO_2_ NPs were purchased from US Research Nanomaterials, Inc. with 30–50nm size for comparative biological analysis. These NPs are called as CH-MnO_2_ NPs.

### Collection of the Plant Material

The fresh *V. betonicifolia* leaves were collected from the surrounding areas of Lahore, Pakistan. Dr. Zaheer-ud-Din Khan (Department of Botany, GC University, Pakistan) made its identification. The voucher specimen (*V. betonicifolia*: GC. Herb. Bot. 213) of plant was deposited at the herbarium of the Department of Botany, GC University, Lahore, Pakistan, and further study approval is not required as per regional guidelines.

### Preparation of Leaves Extract of *Viola betonicifolia*

**Figure 1:**
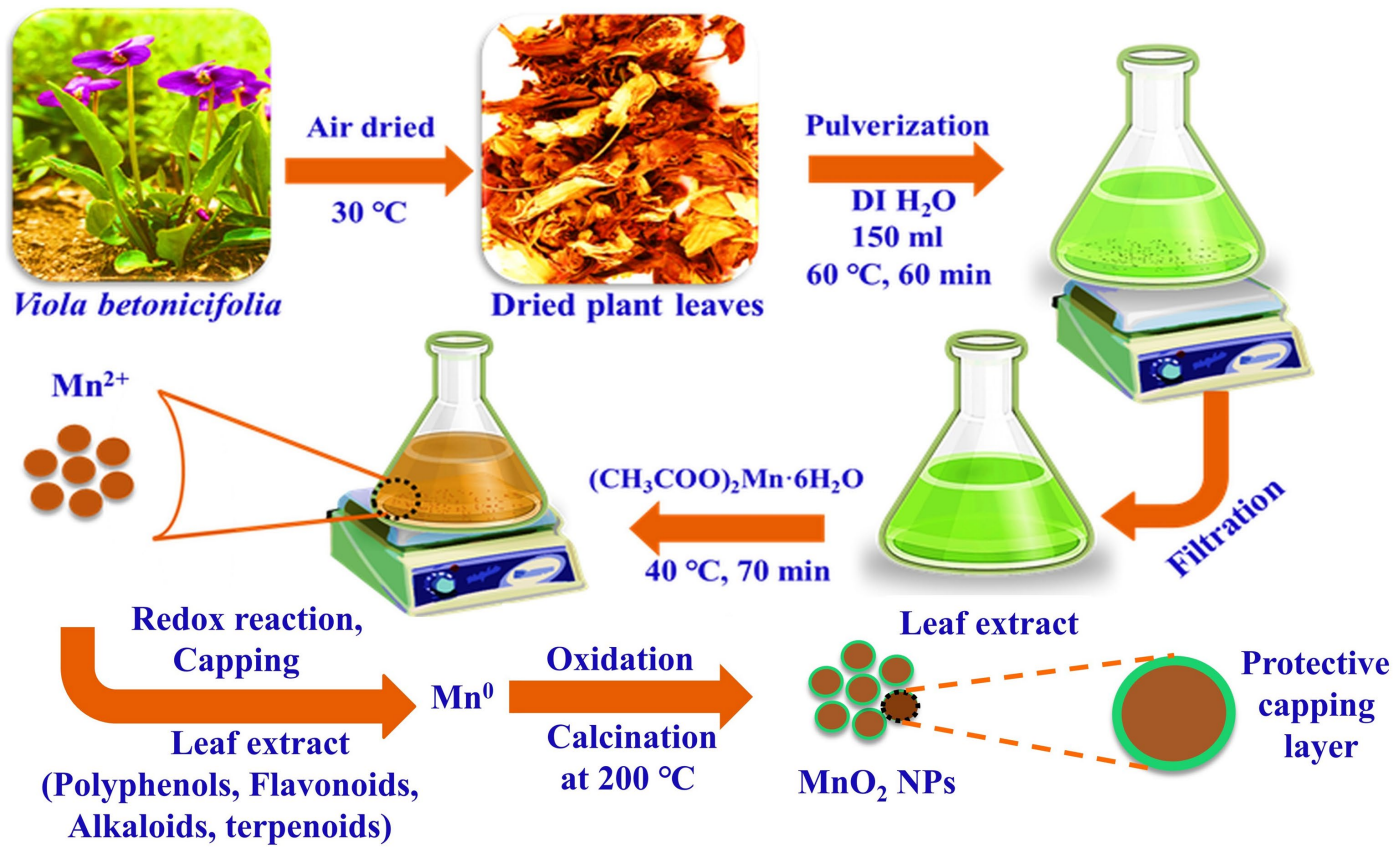
Schematic demonstration and possible synthesis mechanism for the green synthesized VBLE-MnO_2_ NPs with the leaves extract of *Viola betonicifolia*.

The leaves extract of *V. betonicifolia* was prepared by taking 20g of fresh leaves of *V. betonicifolia*. The leaves were washed thoroughly with deionized (DI) water to remove any impurities and dust and air-dried at 30°C. The dried leaves were cut into small pieces, pulverized with the help of a commercial blender, and the resulting plant’s leaves powder was transferred to a 500ml beaker. The 150ml of DI water was then added and stirred at 60°C for 60min. After, the obtained leaves extract of *V. betonicifolia* was cooled down to room temperature and then filtered. The filtrate was collected and stored at 4°C in an airtight glass bottle for further use (Figure 1).

### Biogenic Synthesis of Manganese Dioxide NPs (VBLE-MnO_2_ NPs)

For the biogenic synthesis of manganese dioxide NPs, 1 mm of Manganese acetate was added to 25ml of leaves extract of *V. betonicifolia* (Figure 1). The resulting mixture was heated at 40°C for 70min with continuous stirring at pH 7.15. After, the synthesized manganese dioxide NPs were separated by centrifugation at 3000rpm for 30min from the reaction mixture. After centrifugation, the obtained NPs were washed with DI/ethanol three times and then dried in an oven at 40°C and further calcined in a muffle furnace at 200°C for 3h. Finally, the green synthesized manganese dioxide NPs were stored in a glass bottle for further characterization and named as VBLE-MnO_2_ NPs.

### Characterization

#### X-ray Diffraction

The crystalline nature and phase purity of the green synthesized VBLE-MnO_2_ NPs were determined using the powder X-ray diffraction spectroscopy (XRD), which was carried out at a wavelength (λ) of 0.154nm with LYNXEYE XE-T detector (Haidian, Beijing, China) in a Bruker D2 PHASER. The XRD spectra were recorded in the 2θ range 20–60°, with a scanning rate of 1°/min and a slit width of 6.0mm.

#### Energy-Dispersive X-ray Spectroscopy

The chemical composition of synthesized VBLE-MnO_2_ NPs was performed with an energy-dispersive X-ray (EDX) spectroscopy and Thermo Fisher Scientific Ultradry (Madison, WI, United States) which was attached with SEM.

#### Transmission Electron Microscope

Transmission electron microscopy (TEM) images of the green synthesized VBLE-MnO_2_ NPs were obtained using a Tecnai F 12 microscope (FEI/Philips Tecnai 12 BioTWIN, Baltimore, MD, United States) operating at an acceleration voltage of 200kV (Devi et al., 2019; Khan et al., 2021b). For TEM analysis, the samples were mixed in methanol and then sonicated at 25–30°C. Then, specimens were deposited into a carbon-coated copper grid. After removing the excess solution, the copper grid was left to dry for 5–10min.

#### Zetasizer Dynamic Light Scattering

The stability and the particle size distribution of the fabricated VBLE-MnO_2_ NPs were measured at 25–30°C, with a particle size analyzer (Malvern Zetasizer Nano ZS, Worcestershire, WR14 1XZ, United Kingdom; Devi et al., 2019; Khan et al., 2021b).

### Antibacterial Propensity

The antibacterial propensity of the synthesized VBLE-MnO_2_ NPs was evaluated on *Klebsiella pneumoniae* ATCC^®^700603^™^ and *Staphylococcus aureus* ATCC^®^23235^™^, by using serial dilution method (Khan et al., 2021a). In general, the strains of bacteria were seeded into separate blood agar plates and then cultured for 24h at 37°C. After several bacterium colonies were grown on the plates, they were diluted with phosphate buffer saline (PBS). Their cell density was maintained to 1×10^8^ colony forming units (CFU) per ml. Following that, 10μl of each bacterial culture was separately added to wells of a 24-well microtiter plate with 1.0ml of Mueller-Hinton broth (MHB). For each well, the final concentration of each bacterium was 1×10^6^CFU/ml. A 50μl of each sample solution at 250μg/ml concentration was then transferred to separate wells and incubated at 37°C for 24 h. The bacterial species were then counted in the wells using the serial dilution plate counting method. The antibacterial propensity was expressed in the form of log_10_ reduction in bacterial growth and % killing using the following formulas:


$$\log_{10}\;\mathrm{reduction}=\log_{10}\left({\mathrm{CFU}}_{B}\right)-\log_{10}\left({\mathrm{CFU}}_{A}\right)$$

$$\%\mathrm{killing}=\left({\mathrm{CFU}}_{B}-{\mathrm{CFU}}_{A}\right)/{\mathrm{CFU}}_{B}\times 100$$

here, CFU_B_ and CFU_A_ are the CFU of bacterial strains before and after 24h of incubation, respectively, with the treatment of sample solutions.

#### Live/Dead Bacteria Staining Assay

Live and dead bacterial staining assay was carried out using a confocal laser scanning microscope (CLSM, FV-1200, Olympus, Tokyo, Japan) to confirm the antibacterial activity of green synthesized VBLE-MnO_2_ NPs. The assay was performed following the methods reported by Choi et al. (2017). Briefly, two nucleic dyes, Hoechst 33342 (membrane-permeant) and propidium iodide (PI; membrane-impermeant), were used for staining the live (green) and dead (red) bacteria, respectively. Each bacterium was cultured in nutrient broth in an orbital shaker for 24h at 37°C to reach the stationary phase, which was consists of approximately 10^5^–10^6^ colony forming units (CFU) per ml. After incubation, each bacteria strain was inoculated into sterilized cover glass coated with poly-L-lysine in a 24-well plate and then incubated for 1h for bacterial cells attached to the cover glass. The suspended bacterial cells were then discarded, and each cover glass was gently rinsed three times with a saline solution. For the treatment, each bacterium cells on the cover glass were incubated with green synthesized VBLE-MnO_2_ NPs (250μg/ml) and then incubated for 24h at 37°C. Bacteria cells on cover glass were then stained with an alive and dead bacterial viability kit in accordance with the manufacturer’s recommendations. Dead and live bacterial cells were analyzed with CLSM using an excitation wavelength of 493nm and 350 for PI and Hoechst 33342 and an emission wavelength of 636nm and 461nm for PI and Hoechst 33342, respectively. We only considered green synthesized VBLE-MnO_2_ NPs for live/dead staining assay as they presented excellent antibacterial properties in terms of Log_10_ reductions.

#### ROS Generation Investigation in the Nanoparticles-Treated Bacteria

The CellROX^®^Green staining was further used to examine the death of bacterial species caused by intracellular ROS production. In brief, bacterial species (*K. pneumoniae* and *S*. *aureus*) at 1×10^7^CFU/ml with 70μl of produced VBLE-MnO_2_ NPs at a concentration of 250μg/ml and incubating at 37°C for 24h. Following that, the microbial cells were treated for an additional 30min at 37°C with CellROX^®^Green (5μm). Following that, CLSM was utilized to acquire CLSM images at 485nm absorption and 520nm emission wavelengths. To assess microbial cells’ ability to generate reactive oxygen species (ROS), the results of cells treated with NPs were compared to those treated with 1mm H_2_O_2_ (positive control) and untreated cells (negative control).

### Antifungal Activity

The antifungal activity of the green synthesized VBLE-MnO_2_ NPs was estimated on three mycological species, which include *A. fumigatus* (ATCC^®^13073^™^), *Trichoderma harzianum* (ATCC^®^32086^™^), and *A. flavus* (ATCC^®^9643^™^). A same antibacterial activity method as stated before in section 3.5 was performed, but seeding was performed using a Sabouraud-gentamicin-chloramphenicol (SGC) fungus agar plate and the incubation temperature was maintained at 30°C. The antifungal activity was expressed in the form of log_10_ reduction in bacterial growth and % killing using the following formulas:


$$\log_{10}\;\mathrm{reduction}=\log_{10}\left({\mathrm{CFU}}_{B}\right)-\log_{10}\left({\mathrm{CFU}}_{A}\right)$$

$$\%\mathrm{killing}=\left({\mathrm{CFU}}_{B}-{\mathrm{CFU}}_{A}\right)/{\mathrm{CFU}}_{B}\times 100$$

here, CFU_B_ and CFU_A_ are the CFU of mycological strains before and after 24h of incubation, respectively, with the treatment of sample solutions.

### Anticancer Activity

The anticancer activity of the green synthesized VBLE-MnO_2_ NPs was determined against the MCF-7 breast cancer cells following the MTT [3-(4,5-dimethylthiazol-2-yl)-2,5-diphenyltetrazolium bromide] colorimetric protocol. The MCF-7 cancer cells were kept in Dulbecco’s Modified Eagle’s Medium (DMEM) in an incubator which was set at 5% CO_2_, 95% air, and 37°C. To obtain cell confluency up to 5×10^8^ cells/well, the MCF-7 cells were grown for 24h at 37°C in 100μl of DMEM in a 96-well plate. After 50μl of VBLE-MnO_2_ NPs, plant extract and CH-MnO_2_ NPs at a concentration of 1, 10, 20, 40, 60, 80, 100, and 120μg/ml were added in each well separately containing cultured MCF-7 cells, and the plate was further incubated for 24h at 37°C. The plate was then centrifuged to remove the supernatant and rinsed with PBS solution. A total of 15μl of MTT labeling agent (0.5mg/ml) was then poured into each well; the plate was then put in an incubator for 4h at 37°C. 150μl of DMSO was added to each well to solubilize the undissolved crystals of formazan. The absorption maxima of formazan product in each well were measured at 570nm using a Varian Eclipse spectrophotometer. The percentage of cell viability was calculated using the following formula with the help of the following equation:


$$\%\mathrm{Cell viability}={\mathrm{OD}}_{\mathrm{sample}}/{\mathrm{OD}}_{\mathrm{control}}\times 100$$

#### Live and Dead Staining Analysis

Further detection of cell viability utilizing the live and dead staining kit was investigated with the fluorescent staining technique. As already described above, the same experiments were repeated until MCF-7 cancer cells were treated with various samples concentration (10μl of 120μg/ml) and subsequently incubated. After incubation, staining solution at a 4 μg/ml concentration was added to each well and then incubated for 20min at 37°C. Dead and live MCF-7 cells were analyzed with CLSM using an excitation wavelength of 493nm and 350 for PI and Hoechst 33342 and an emission wavelength of 636nm and 461nm for PI and Hoechst 33342, respectively.

### Antioxidant Activity in Terms of Linoleic Acid (%) Inhibition

The antioxidant activity of the green synthesized VBLE-MnO_2_ NPs was evaluated according to the linoleic acid (%) inhibition method reported by Iqbal et al. (2005). For this purpose, 100μg/ml concentration of each sample (green synthesized VBLE-MnO_2_ NPs, plant extracts, and chemically synthesized VBLE-MnO_2_ NPs) was added to the solution mixture of 99.99% ethanol (10ml), 0.2M sodium phosphate buffer (pH 7.0, 10ml), and linoleic acid (0.13ml). With DI, the total amount of the resultant mixture was increased to 25ml and then incubated for 360h at 40°C. The thiocyanate technique was used to assess the extent of oxidation. Each sample solution was diluted by adding 0.2ml of each sample solution in 10ml of ethanol (75%). Then, 0.2ml FeCl_2_ (20mm in 3.5% HCl) and 0.2ml of aqueous ammonium thiocyanate solution (30%) were added, and then, the mixture was mixed for 3min. Measurements of the absorption maxima were made at 500nm wavelength. The percentage inhibition (% Inhibition) of linoleic acid was determined using the formula as:


$$\%\mathrm{Inhibition}=100-\left[\begin{array}{l} \left(\mathrm{Absorbance of sample}\right)/\\ \left(\mathrm{absorbance of control}\right)\times 100 \end{array}\right]$$

Ascorbic acid was used as an external standard, and linoleic acid was used as a control without any treatment.

### Cytobiocompatibility Analysis

The cytobiocompatibility of the green synthesized VBLE-MnO_2_ NPs against the hMSC cell line in comparison with the CH-MnO_2_ NPs and plant extract was evaluated *via* the MTT protocol as reported by Emam et al. (2017). The hMSC cells were kept in Dulbecco’s Modified Eagle’s Medium (DMEM) in an incubator which was set at 5% CO_2_, 95% air, and 37°C. To obtain cell confluency up to 5×10^8^ cells/well, the hMSC cells were grown for 24h at 37°C in 100μl of DMEM in a 96-well plate. After 50μl of green synthesized VBLE-MnO_2_ NPs, plant extract and CH-MnO_2_ NPs at a concentration of 120μg/ml were added in each well separately containing cultured hMSC cells, and the plate was further incubated for 24h at 37°C. The plate was then centrifuged to remove the supernatant and rinsed with PBS solution. A total of 15μl of MTT labeling agent (0.5mg/ml) was then poured into each well; the plate was then put in an incubator for 4h at 37°C. A total of 150μl of DMSO was added to each well to solubilize the undissolved crystals of formazan. The optical density (OD) of formazan product in each well was measured at 570nm using a Varian Eclipse spectrophotometer. The percentage of cell viability was calculated using the following formula:


$$\%\mathrm{Cell viability}={\mathrm{OD}}_{\mathrm{sample}}/{\mathrm{OD}}_{\mathrm{control}}\times 100$$

### Statistical Analysis

All trials were conducted in triplicate, and the findings are given as mean±standard deviation. To ascertain the statistical significance, we used ANOVA with a predetermined significance level (0.05).

## Results and Discussion

### Synthesis Mechanism

For the fabrication of VBLE-MnO_2_ NPs, the leaf extract of *V. betonicifolia* was utilized as a reducing and capping agent. The formation of VBLE-MnO_2_ NPs was visually tracked by observing the color change caused by the addition of a precursor to leaf extract. The reaction mixture’s color shift from yellowish green to brownish indicated the formation of the required manganese dioxide NPs. This color shift occurred as a consequence of the nanoparticle’s surface plasmon resonance action. Several reports demonstrate that the leaves extract of *V. betonicifolia* is a rich source of several biogenic phytomolecules, including alkaloids, flavonoids, tannins, phenolic compounds, saponins, and triterpenoids [14–17]. During the biosynthesis process, these phytomolecules might be functioned as reducing the manganese ions to zero-valent species *via* reduction and oxidation reaction with the production of keto form products. Further, other secondary metabolites (surfactants, proteins, alkaloids, etc.) present in the leaf extract of the *V. betonicifolia* simultaneously stabilized and capped the zero-valent species of Mn^0^. During air-drying and calcination at 200°C, zero-valent species of Mn^0^ would be readily oxidized and converted into MnO_2_ nanoparticles capped with phytomolecules of plant leaf extract (Figure 1). Similar green synthesis mechanism of NPs using different plant extract was also reported by Dzul-Erosa et al. (2018); Khalafi et al. (2019); Rafique et al. (2019); Ciorîță et al. (2020); Gurgur et al. (2020); López and Antuch (2020); Khan et al. (2020b).

### Characterization

**Figure 2:**
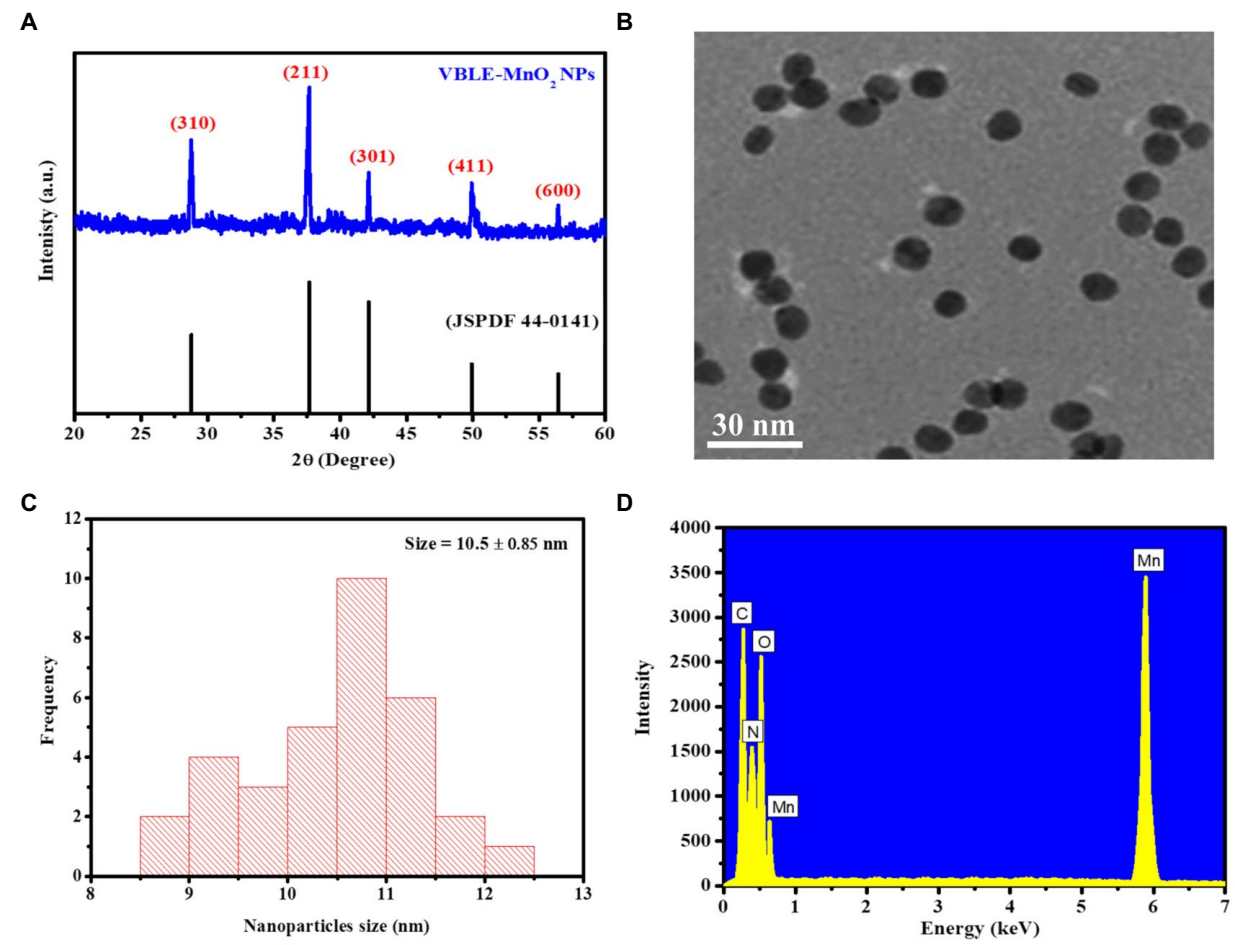
**(A)** XRD pattern, **(B)** TEM image, **(C)** size distribution, and **(D)** EDX pattern for the green synthesized VBLE-MnO_2_ NPs.

XRD analysis was carried out to analyze the crystallinity of the VBLE-MnO_2_ NPs synthesized using the leaves extract of *V. betonicifolia*. Figure 2A shows an XRD pattern of the VBLE-MnO_2_ NPs. XRD pattern demonstrates five distinguish peaks at 2θ=28.78°, 37.66°, 42.14*°*, 49.90°, and 56.44°, indexed to (310), (211), (301), (411), and (600) crystal plane of VBLE-MnO_2_ NPs (JSPDF 44-0141; He et al., 2018, 2019; Wan et al., 2019). Moreover, the XRD pattern indicates that the VBLE-MnO_2_ NPs are highly crystalline, as evident from the intensity of the peaks. Figure 2B presents the TEM images of VBLE-MnO_2_ NPs. The TEM image depicts that the synthesized VBLE-MnO_2_ NPs are spherical with homogeneous dispersity. The particle size of the synthesized VBLE-MnO_2_ NPs was estimated to be 10.5±0.85nm using Zetasizer Dynamic Light Scattering, as shown in Figure 2C. To determine the chemical composition of the VBLE-MnO_2_ NPs, the EDX analysis was then conducted. Figure 2D shows the EDX pattern. EDX spectra indicate four characteristics peaks corresponding to carbon, nitrogen, oxygen, and manganese at 0.27kev, 0.39kev, 0.52kev, and 5.8kev, respectively. A small peak at 0.63kev attribute to Mn is also evident in EDX spectra (Panahi-Kalamuei et al., 2016; Srither et al., 2016; Ogunyemi et al., 2019). The EDX peaks (C and N) might be attributed to the adsorption of secondary metabolites from *V. betonicifolia* leaves extract on the surface of VBLE-MnO_2_ NPs (Ciorîță et al., 2020). Thus, these characterization findings confirmed the effective synthesis of VBLE-MnO_2_ NPs using a leaf extract of *V. betonicifolia*.

### Antibacterial Activity

**Figure 3:**
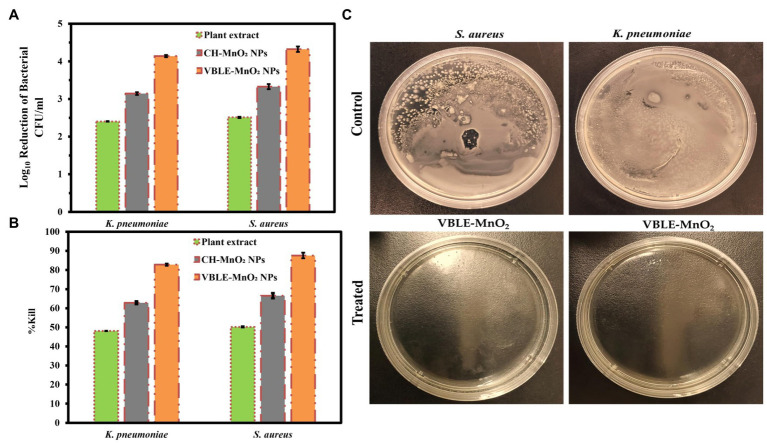
Antibacterial activity of VBLE-MnO_2_ NPs in terms of **(A)** log_10_ reduction and **(B)** % killing efficiency of bacterial strains in comparison with leaves extract of *Viola betonicifolia* and CH-MnO_2_ NPs. **(C)** Representative images of control and treated *S. aureus* and *K. pneumonia* with VBLE-MnO_2_.

Antibacterial activity of VBLE-MnO_2_ NPs was determined in terms of log_10_ reduction and % killing efficiency of bacterial strains compared to leaves extract of *V. betonicifolia* and CH-MnO_2_ NPs. The results are presented in Figures 3A–C. The outcomes have been shown that the synthesized VBLE-MnO_2_ NPs displayed 4.14±0.03 and 4.65±0.07 log_10_ reductions in CFU of *K. pneumoniae and S. aureus*, respectively, with more than 80% killing efficiency. On the other hand, CH-MnO_2_ NPs demonstrated the least antibacterial activity (log_10_ reductions 3.14±0.04 and 3.33±0.08 against *K. pneumoniae and S. aureus*, respectively) than VBLE-MnO_2_ NPs, as shown in Figures 3A,B. It is worth noting that the leaves extract of *V. betonicifolia* has shown significant antibacterial behavior (log_10_ reductions >2.35) and more than 45 percent killing performance against all bacteriological species tested. This indicates that the leaves extract of *V. betonicifolia* possesses pharmacologically important phytomolecules capable of killing bacterial strains effectively (Feyzabadi et al., 2017; Rizwan et al., 2019). Additionally, we conducted an ANOVA test on the antibacterial findings, which showed a significant difference of *p*<0.005.

#### LIVE and DEAD Staining Assay

**Figure 4:**
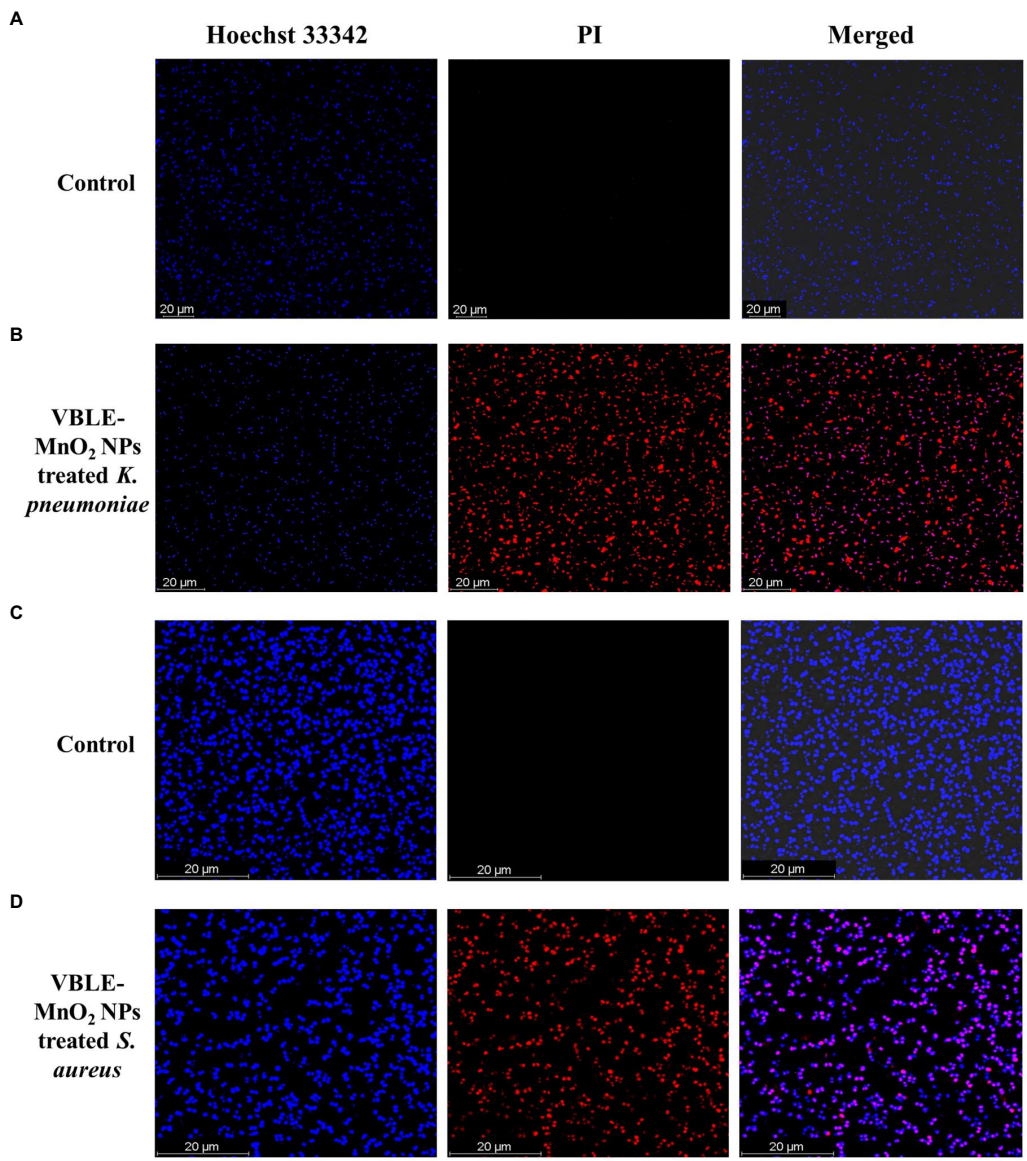
CLSM images of untreated [control **(A,C)**] and treated **(B,D)** bacteria with synthesized VBLE-MnO_2_ NPs. Red represents dead bacterial cells.

LIVE and DEAD staining assay was used to evaluate the interaction of synthesized VBLE-MnO_2_ NPs with cells and subsequent cell death upon labeling with Hoechst 33342 and PI. The permeability of the bacterial membrane to these dyes is dependent on the cellular membrane’s potential, which allows for the differentiation of alive and dead cells. Hoechst 33342 is a membrane-permeant dye can stain both alive and dead cells by interlacing DNA, while PI is a membrane-impermeant dye that permeates only through dead cells’ perforated membranes and can stain only dead cells (Arakha et al., 2015; Ramalingam et al., 2016). As illustrated in Figures 4A,C, the untreated *K. pneumoniae and S. aureus*, stained only with Hoechst 33342, suggesting that they were intact and alive. At the same time, VBLE-MnO_2_ NPs treated cells fluoresced red (Figures 4B,D). This revealed that VBLE-MnO_2_ NPs triggering cell death had an effect on the integrity and permeability of the cell membrane.

#### Reactive Oxygen Species Generation in Bacteria Treated With VBLE-MnO_2_

**Figure 5:**
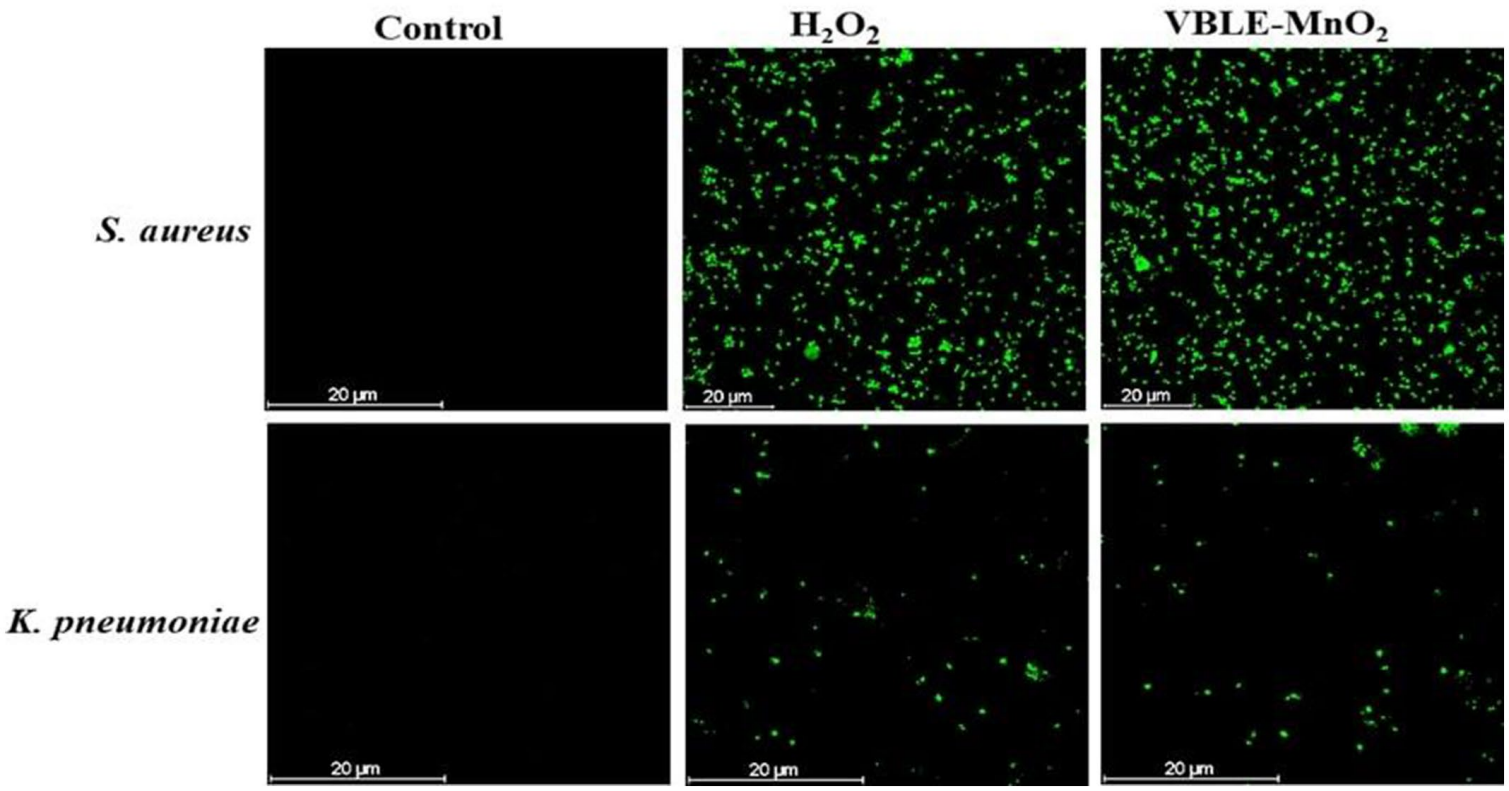
CLSM images of ROS generation in untreated (control) and treated bacteria with hydrogen peroxide (H_2_O_2_) and synthesized VBLE-MnO_2_.

Oxidative stress caused by intracellular ROS production has been shown to destroy microbial strains (Kim et al., 2007). Metal nanoparticles (NPs) interact with bacteria to produce ROS, which can lead to oxidative stress inside the cell and the destruction of organelles and biomolecules. Figure 5 depicts the results of the CellROX^®^Green test, which was used to assess oxidative stresses in microbial cells following treatment with VBLE-MnO_2_ NPs. No intracellular ROS species were produced in either bacterial cell under control. Both bacteria treated with VBLE-MnO_2_ produced ROS comparable to that produced by H_2_O_2_. These findings suggest that one explanation for the outstanding antibacterial activity of the VBLE-MnO_2_ produced is the production of ROS, which causes bacterial cells to die. Graphical presentation of ROS production mechanism in bacterial cells due to VBLE-MnO_2_ NPs is shown in Figure 5.

#### Antibacterial Mechanism

**Figure 6:**
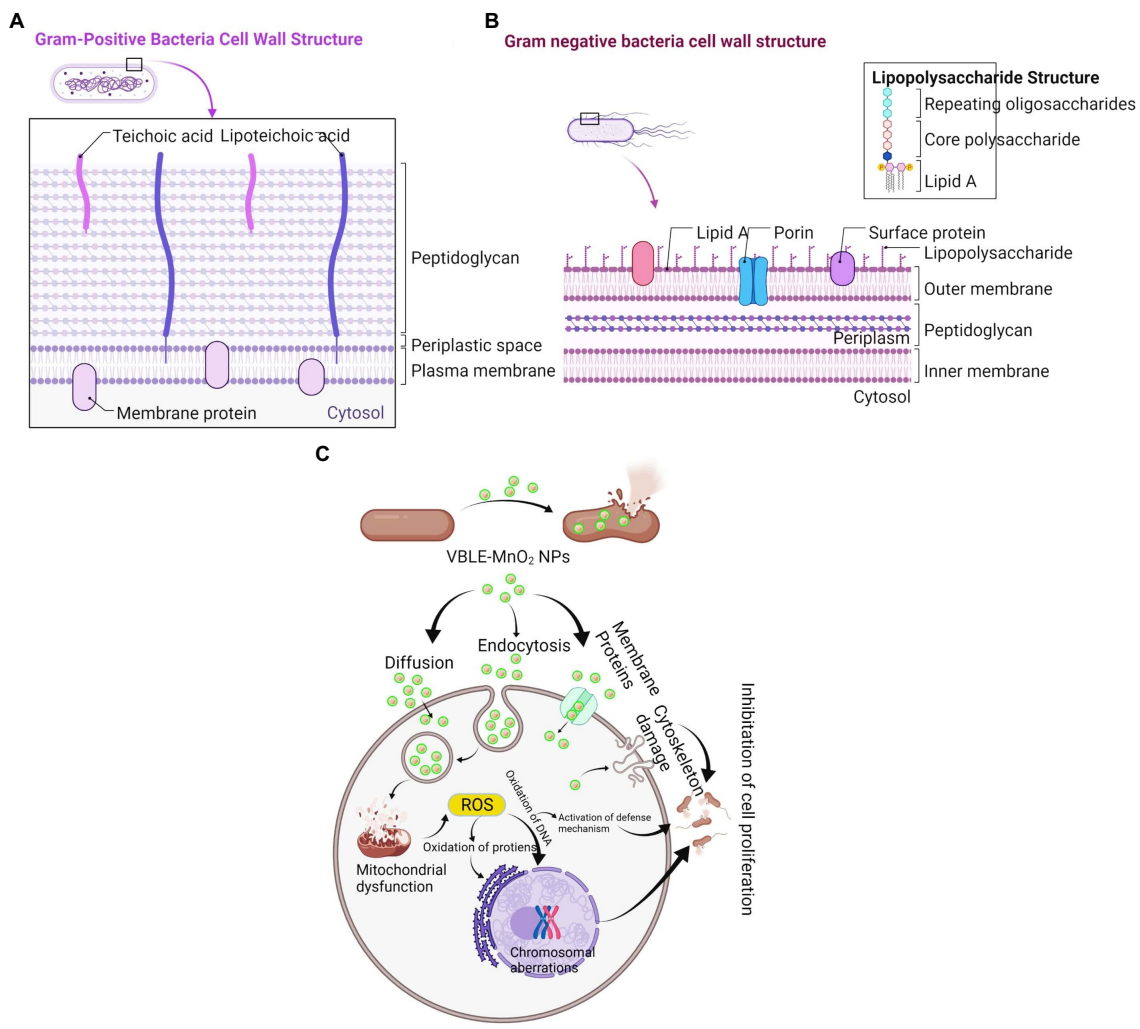
Comparison of **(A)** Gram-positive and **(B)** Gram-negative bacterial cell wall. **(C)** Proposed antibacterial mechanism of green synthesized VBLE-MnO_2_ NPs.

Additionally, the superior antibacterial action of VBLE-MnO_2_ NPs can be due to the synergy influence of the nanoparticle’s physical characteristics and the adsorption of biologically active phytomolecules from the leaves extract of *V. betonicifolia* on their surface (Huang et al., 2011; Khan et al., 2018, 2020a,b, 2021a,b). The results were further demonstrated that the synthesized VBLE-MnO_2_ NPs appeared more active toward the Gram-positive in contrast to that of Gram-negative bacteriological species. This may be due to structural and compositional variations between Gram-negative and Gram-positive bacterial strains’ cell walls (Figures 6A,B; Bindhu and Umadevi, 2014; Muthukumar et al., 2016; Boomi et al., 2019). Manjula et al. and Kunkalekar et al. were also reported the same more inhibitory effect of MnO_2_ NPs toward Gram-positive than Gram-negative bacteria (Kunkalekar et al., 2013; Manjula et al., 2019).

Numerous reports have shown that the antibacterial effect of nanomaterials is mostly due to physical (e.g., lipid molecule disintegration) and chemical (e.g., oxidative stress) deterioration (Du et al., 2020). The synergy of antibacterial activity was defined in this study using a two-step approach: (1) generation of reactive oxygen species (Figures 5, 6C) and (1) membrane damage, leakage of electrolytes and intracellular contents, and decrease in ATPase activity, all of which contribute to bacterial death (Figures 4, 6C).

### Antifungal Activity

**Figure 7:**
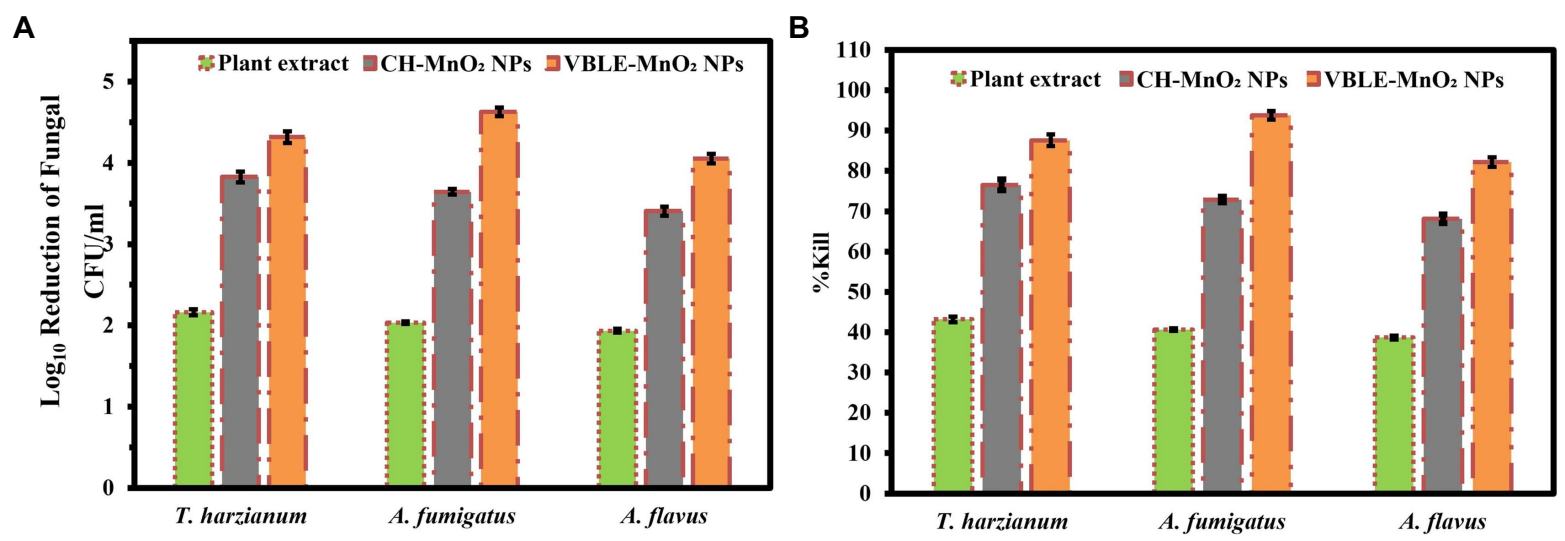
Antifungal activity of VBLE-MnO_2_ NPs in terms of **(A)** log_10_ reduction and **(B)** % killing efficiency of fungal strains in comparison with leaves extract of *V. betonicifolia* and CH-MnO_2_ NPs.

In contrast to leaves extract of *V. betonicifolia* and CH-MnO_2_ NPs, the antifungal activity of synthesized VBLE-MnO_2_ NPs was determined in terms of log_10_ reduction and % killing efficiency of mycological strains. Figures 7A,B illustrate the findings. The results indicated that the synthesized VBLE-MnO_2_ NPs significantly reduced the CFU of A. flavus, T. harzianum, and A. fumigatus by 4.05±0.06, 4.32±0.07, and 4.63±0.05 log_10_ reductions, respectively, with a killing efficiency of more than 82 percent. CH-MnO_2_ NPs, on the other hand, demonstrated significantly lower antifungal activity than VBLE-MnO_2_ NPs, as shown in Figures 7A,B. Furthermore, we conducted an ANOVA test on the antifungal activity results, which exhibited a significant difference of *p*<0.002. It is worth noting that the leaves extract of *V. betonicifolia* also demonstrated substantial antifungal activity and percent killing efficiency against all tested mycological strains. This indicates that the *V. betonicifolia* leaf extract contains biologically active phytomolecules that are particularly effective at destroying fungal strains (Katoch et al., 2017). Additionally, the superior antifungal activity of VBLE-MnO_2_ NPs can be due to the synergistic impact of the nanoparticle’s physical properties and the adsorbed biologically active phytomolecules from the leaves extract of *V. betonicifolia* on their surface. The similar antifungal results were also reported with the green synthesized MnO NPs using leaf extract of *Abutilon indicum* by Khan et al. (2020b).

### Biofilm Inhibition Investigations

**Figure 8:**
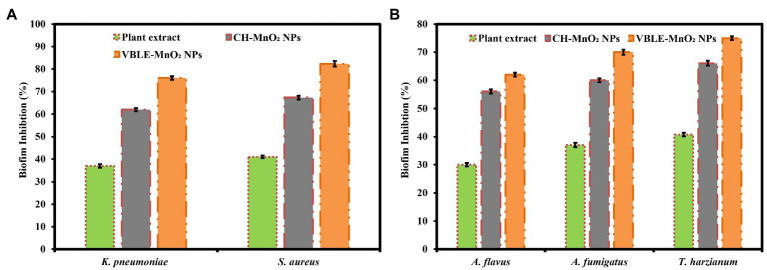
Biofilm inhibition performance of the synthesized VBLE-MnO_2_ NPs against the **(A)** bacterial and **(B)** fungal strains in comparison with *V. betonicifolia* leaves extract and CH-MnO_2_ NPs.

The biofilm inhibition activity of VBLE-MnO_2_ NPs was evaluated against infectious bacterial and mycological species in contrast to *V. betonicifolia* leaves extract and CH-MnO_2_ NPs. Figures 8A,B illustrate the findings. The findings indicate that the VBLE-MnO_2_ NPs exhibited substantial biofilm inhibitory activity, inhibiting the production of biofilms of both bacterial and mycological strains. Although CH-MnO_2_ NPs inhibited the production of biofilms in both microbial species but were less than VBLE-MnO_2_ NPs synthesized with *V. betonicifolia* leaves extract. Moreover, the *V. betonicifolia* leaves extract also demonstrated good biofilm inhibition performance, as evident from Figures 8A,B. In addition, we performed an ANOVA test on the biofilm inhibition activity data against both of fungal and bacterial strains, which revealed a significant difference of *p*<0.002 and *p*<0.001, respectively. The remarkable biofilm inhibition efficiency of the synthesized VBLE-MnO_2_ NPs may be a result of the synergy between their physical properties and the incorporation of phytomolecules from *V. betonicifolia* leaves extract on the nanoparticle’s surface.

### Cytotoxic Potential Against MCF-7 Carcinoma Cells

**Figure 9:**
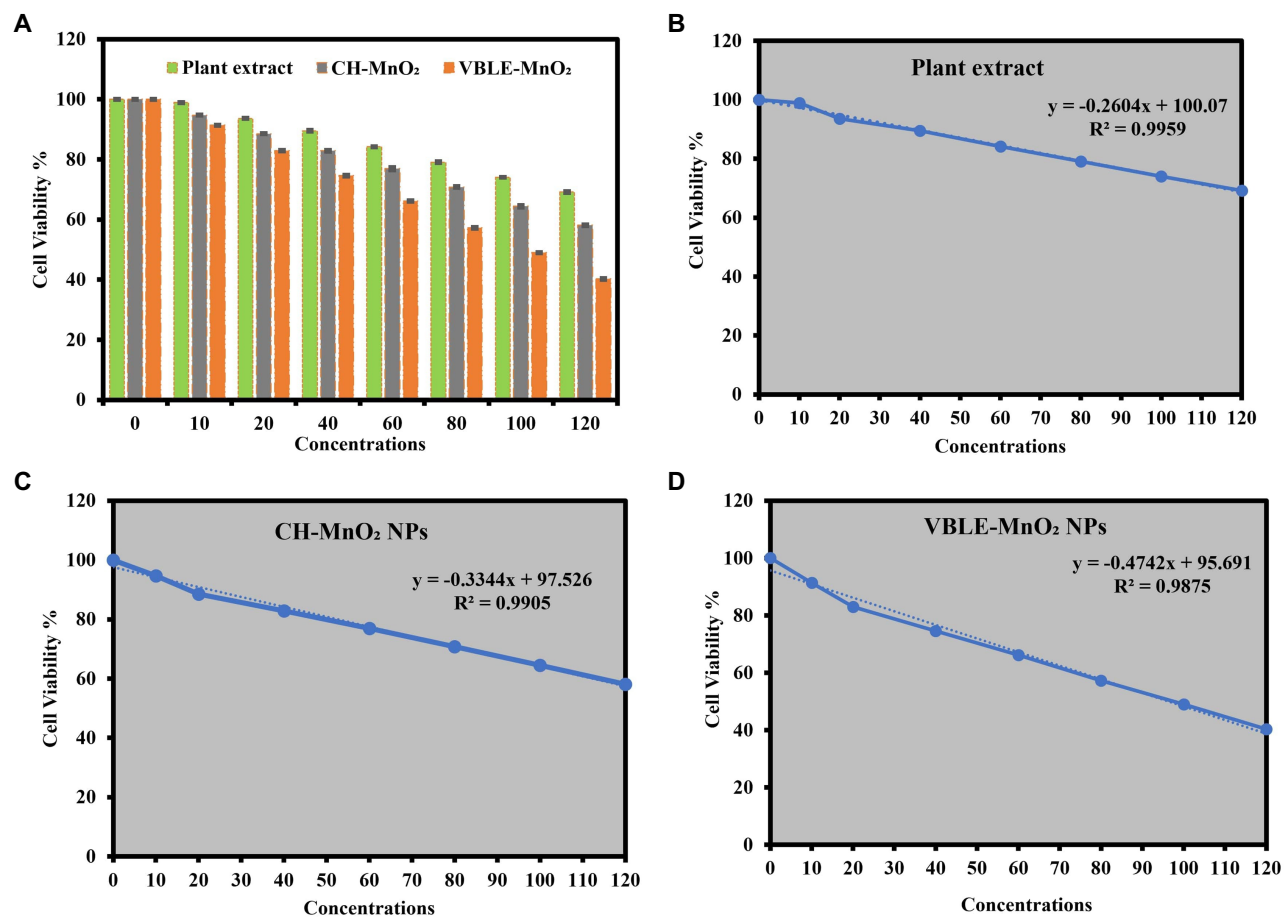
**(A)** Cytotoxic potential in terms of cell viability percentage against MCF-7 carcinoma cells treated with *V. betonicifolia* leaves extract, CH-MnO_2_ NPs, and VBLE-MnO_2_ NPs. Linear plot and regression coefficient between the cell viability % of the MCF-7 carcinoma cells with different concentrations of **(B)** *V. betonicifolia* leaves extract, **(C)** CH-MnO_2_ NPs, and **(D)** VBLE-MnO_2_ NPs.

The cytotoxic potential of VBLE-MnO_2_ NPs was determined by comparing them to *V. betonicifolia* leaves extract and CH-MnO_2_ NPs. The findings indicated that all samples exhibited dose-dependent therapeutic effectiveness (Figure 9A). The maximum inhibitory effects on MCF-7 melanoma cells were observed when all samples were concentrated to 120μg/ml. The VBLE-MnO_2_ NPs displayed excellent cytotoxic activity in comparison with *V. betonicifolia* leaves extract and CH-MnO_2_ NPs at all dose levels. The extraordinary cytotoxic activity of the produced VBLE-MnO_2_ NPs might be attributed by a synergy between their physical characteristics and the inclusion of phytomolecules from *V. betonicifolia* leaves extract on the NP’s surface. It is worth mentioning that the leaves extract of *V. betonicifolia* also exhibited effective cytotoxic activity against MCF-7 carcinoma cells. This shows that the leaves extract of *V. betonicifolia* contains phytomolecules of pharmacological significance capable of efficiently killing cancerous cells. Moreover, linearity has been observed between the cell viability % of the MCF-7 carcinoma cells with different concentrations of *V. betonicifolia* leaves extract, CH-MnO_2_ NPs, and VBLE-MnO_2_ NPs as shown in Figures 9B–D, respectively. We further performed ANOVA test on cytotoxic results of three groups against different concentrations of 0, 10, 20, 40, 60, 80, 100, and 120μg/ml and the results revealed the statistical difference by *p*>0.05, *p*<0.001, *p*<0.003, *p*<0.004, *p*<0.008, *p*<0.001, *p*<0.005, and *p*<0.006, respectively. Our synthesized VBLE-MnO_2_ NPs appeared more active toward killing the carcinoma cells as compared to green synthesized Ag-MnO_2_ NPs previously reported by Ciorîță et al. (2020) but comparable to the NPs reported by Khan et al. (2020b). The similar dose-dependent cytotoxic activity was also reported by Khan et al. (2020b).

#### Live and Dead Staining

**Figure 10:**
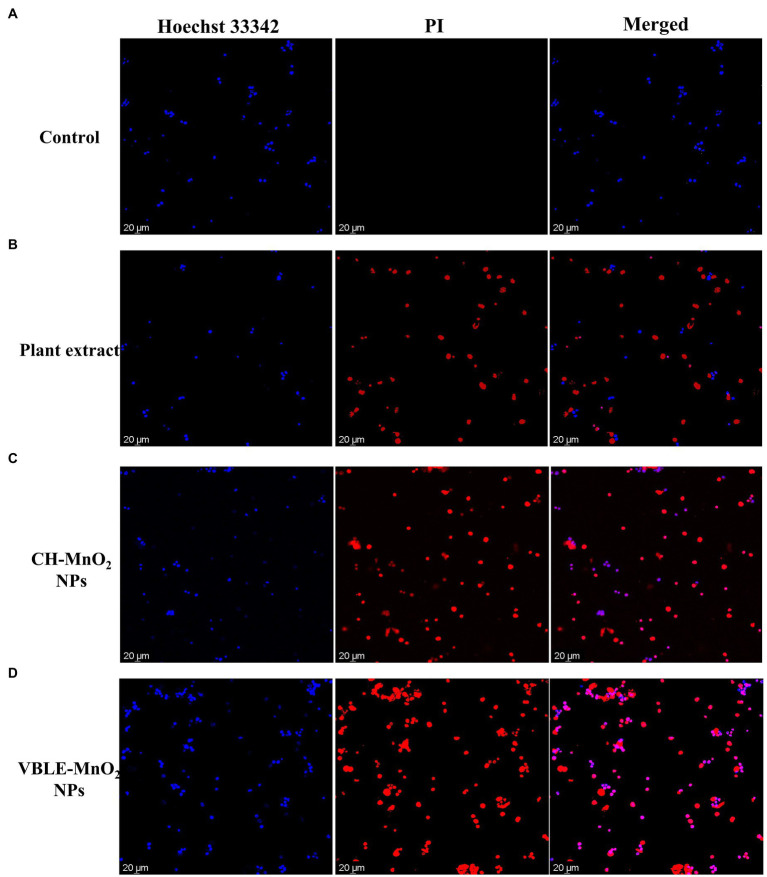
CLSM images of the live and dead MCF-7 cancer cells stained with Hoechst 33342 and PI dye before **(A)** control and after treatment with **(B)** plant extract, **(C)** CH-MnO_2_ NPs, and **(D)** VBLE-MnO_2_ NPs.

CLSM was further used to confirm the cytotoxicity against MCF-7 cancerous cells utilizing the live and dead fluorescence staining experiment. Figures 10A–D exhibit living, and deceased MCF-7 cancerous cells dyed with Hoechst 33342 and PI dye, respectively. Hoechst 33342 is a membrane-permeant dye can stain both alive and dead cells by interlacing DNA, while PI is a membrane-impermeant dye that permeates only through dead cells’ perforated membranes and can stain only dead cells. The findings showed that VBLE-MnO_2_ NPs had the greatest cytotoxic impact on MCF-7 carcinoma cells, killing almost of malignant cells, while CH-MnO_2_ NPs had a moderate toxic impact on MCF-7 cancer cells. It is worth noting that leaf extract was similarly hazardous to MCF-7 cancer cells, suggesting that *V. betonicifolia* leaves extract contains pharmacologically active phytomolecules. These findings are compatible with the findings of MTT analyses.

### Antioxidant Activity

**Figure 11:**
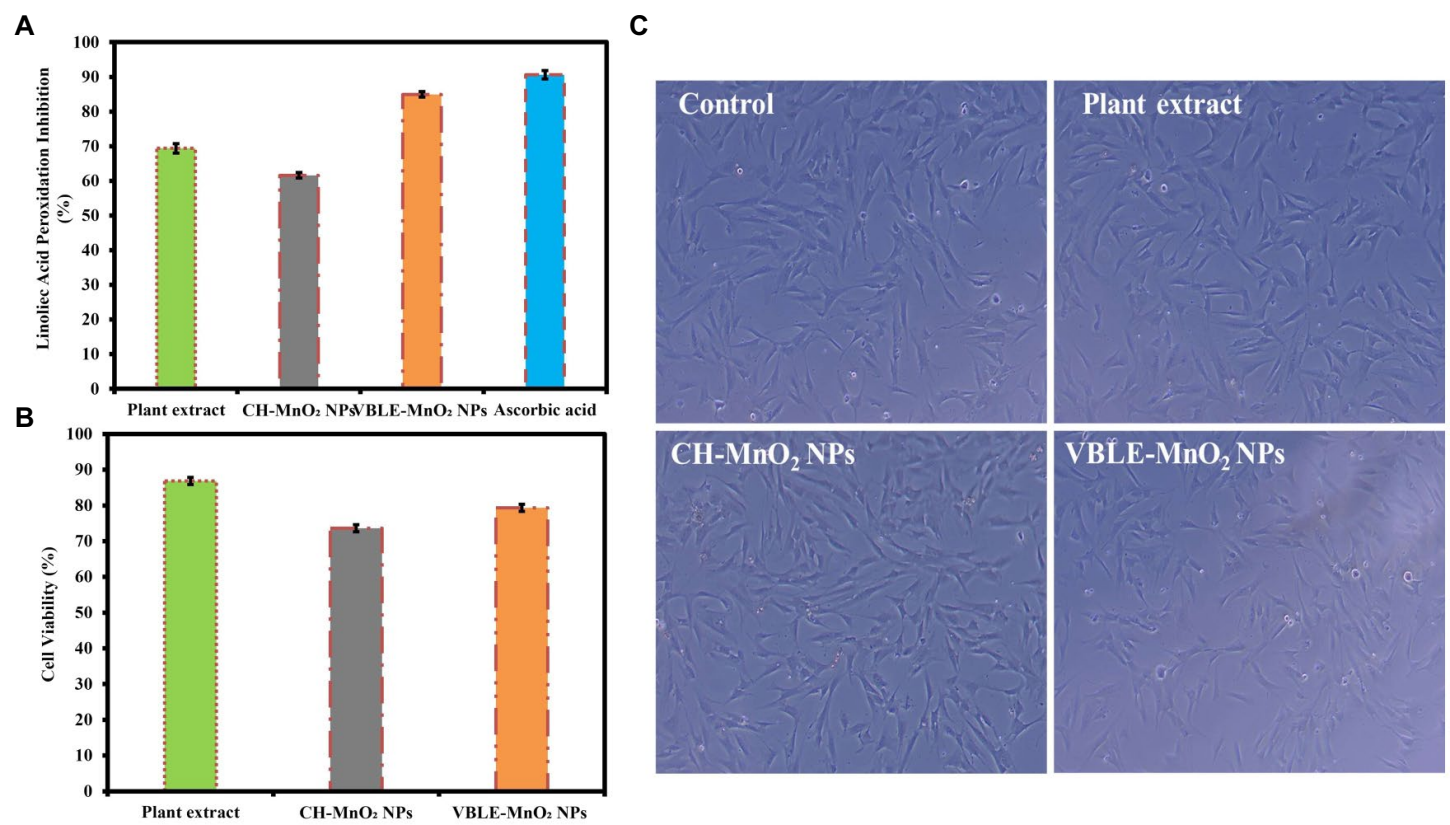
**(A)** Antioxidant activity of the newly synthesized VBLE-MnO_2_ NPs in comparison with *V. betonicifolia* leaves extract, CH-MnO_2_ NPs, and external standard (Ascorbic acid). **(B,C)** Biocompatibility analysis of the newly synthesized VBLE-MnO_2_ NPs with hMSC cells compared to the leaves extract of *V. betonicifolia* and CH-MnO_2_ NPs.

Antioxidant activity of the newly synthesized VBLE-MnO_2_ NPs was investigated in comparison with the leaves extract of *V. betonicifolia*, CH-MnO_2_ NPs, and external standard (ascorbic acid). Figure 11A illustrates the findings. The anti-linoleic acid peroxidation activity of newly synthesized VBLE-MnO_2_ NPs was superior (84.94±0.77%) to that of *V. betonicifolia* leaves extract and CH-MnO_2_ NPs, although a little less than that of ascorbic acid (90.57±1.21%). On the other hand, CH-MnO_2_ NPs exhibited the lowest antioxidant function, exhibiting the lowest percentage of anti-linoleic acid peroxidation (61.61±0.79%). Additionally, the leaves extract of *V. betonicifolia* demonstrated superior antioxidant function by inhibiting linoleic acid peroxidation (69.37±1.37%) as compared to CH-MnO_2_ NPs. These findings suggested that *V. betonicifolia* leaves extract contains a high concentration of natural antioxidants (Muhammad et al., 2013b; Rizwan et al., 2019). Furthermore, the inclusion of phytomolecules from *V. betonicifolia* leaves extract on the nanoparticle’s surface could be responsible for the VBLE-MnO_2_ NPs’ superior antioxidant activity. We performed an ANOVA test on the antioxidant findings and determined that they were statistically significant at *p*<0.004. The similar enhanced antioxidant activity of the green synthesized NPs was also reported by Khan et al. (2020a); Shahid et al. (2021).

### Biocompatibility Evaluation

The biocompatibility of VBLE-MnO_2_ NPs was assessed with the hMSC cells *in vitro* compared to the *V. betonicifolia* leaves extract and CH-MnO_2_ NPs. The results are expressed as a percentage of cell viability, as shown in Figure 11B. The findings indicated that CH-MnO_2_ NPs had the lowest cell viability percentage (73.54±0.82%). On the other side, newly synthesized VBLE-MnO_2_ NPs demonstrated a cell viability percentage (79.33±0.75%) with hMSC cells. Additionally, it is noteworthy to mention that the extract of *V. betonicifolia* leaves contains biocompatible secondary metabolites that demonstrated excellent biocompatibility (cell viability percentage 86.84±0.85%) with hMSC cells. It has been reported that different solvent extracts of *V. betonicifolia* are safe to use and have no toxic effects (Muhammad et al., 2012, 2013c; Rizwan et al., 2019). We used an ANOVA test to determine the statistical significance of the biocompatibility findings and discovered a *p*<0.007 significance level.

We next examined the morphological changes in hMSC cells treated with VBLE-MnO_2_ NPs, plant extract, and CH-MnO_2_ NPs at a 120μg/ml concentration using an inverted microscope. The inverted micrograph of hMSC cells is shown in Figure 11C. The photographs demonstrate that following treatment with plant extract and VBLE-MnO_2_ NPs, the morphology of hMSC cells remained comparable to that of the control (untreated cells). On the other hand, CH-MnO_2_ NPs caused toxicity in hMSC cells, reducing their volume and cytoplasm, and altering their shape. The viability of cells and the inverted microscopy findings were found to be consistent. As a result, it can be inferred that phytomolecules contained in the leaves extract of *V. betonicifolia* may be responsible for the VBLE-MnO_2_ NPs’ enhanced cytobiocompatibility. The similar enhanced biocompatibility of the green synthesized NPs with various normal cell lines was also reported by Ciorîță et al. (2020) and Khan et al. (2020a,b, 2021b).

## Conclusion

The manganese dioxide NPs (VBLE-MnO_2_ NPs) were synthesized using the leaves extract of *V. betonicifolia* very first time, in which the plant’s secondary metabolites function as both reducing and capping agents. The synthesized VBLE-MnO_2_ NPs were successfully characterized with different spectroscopic techniques. The synthesized VBLE-MnO_2_ NPs were investigated for different biological activities (antioxidant, cytotoxicity, antibacterial, antifungal, and biofilm inhibition). The results were demonstrated that the synthesized VBLE-MnO_2_ NPs presented excellent antibacterial, antifungal, and biofilm inhibition performance against all the tested microbial species compared to plant leaves extract and commercially purchased chemically synthesized manganese dioxide NPs (CH-MnO_2_ NPs). Moreover, they also exhibited significant antioxidant potential, which was comparable to the external standard; however, it was higher than plant leaves extract and CH-MnO_2_ NPs. The synthesized CH-MnO_2_ NPs displayed good cytobiocompatibility with hMSC cells compared to CH-MnO_2_ NPs. The enhanced antioxidant, cytobiocompatibility, antibacterial, antifungal, biofilm inhibition, and cytotoxic efficacy of VBLE-MnO_2_ NPs as compared to CH-MnO_2_ NPs might be attributed to the synergistic effect of the nanoparticle’s physical properties and the adsorbed biologically active phytomolecules from the leaves extract of *V. betonicifolia* on their surface. Thus, our work offers a unique environmentally sustainable technique for the manufacture of nanomaterials bestowed with enhanced and/or additional therapeutic properties obtained from their herbal sources. Furthermore, more study should be conducted to determine the efficacy and dose response biocompatibility of VBLE-MnO_2_ NPs in therapeutic interventions. The VBLE-MnO_2_ NPs synthesized in this research could be utilized to provide antibacterial coatings for medical devices, such as catheters, tubing, sensors, and bandages, thereby lowering the incidence of pathogenic bacteriological and mycological infections induced by biomaterials and medical implants.

## Data Availability Statement

The raw data supporting the conclusions of this article will be made available by the authors, without undue reservation.

## Author Contributions

HL and XZ: conceptualization, methodology, software, validation, formal analysis, and investigation. SK, WL, and LW: resources, data curation, writing—original draft preparation, writing—review and editing, visualization, supervision, and project administration. WL and LW: funding acquisition. All authors have read and agreed to the published version of the manuscript.

## Funding

This research was funded by the National Natural Science Foundation of China (81600900) and the Science Foundation of Stomatological Hospital, Southern Medical University (PY2019013), and the Postdoctoral sustentation fund of Shunde district, Foshan city.

## Conflict of Interest

The authors declare that the research was conducted in the absence of any commercial or financial relationships that could be construed as a potential conflict of interest.

## Publisher’s Note

All claims expressed in this article are solely those of the authors and do not necessarily represent those of their affiliated organizations, or those of the publisher, the editors and the reviewers. Any product that may be evaluated in this article, or claim that may be made by its manufacturer, is not guaranteed or endorsed by the publisher.
